# Prospective observational study of bevacizumab combined with paclitaxel as first- or second-line chemotherapy for locally advanced or metastatic breast cancer: the JBCRG-C05 (B-SHARE) study

**DOI:** 10.1007/s12282-020-01138-4

**Published:** 2020-07-26

**Authors:** Yutaka Yamamoto, Hiroyasu Yamashiro, Uhi Toh, Naoto Kondo, Rikiya Nakamura, Masahiro Kashiwaba, Masato Takahashi, Koichiro Tsugawa, Takashi Ishikawa, Takahiro Nakayama, Shoichiro Ohtani, Toshimi Takano, Tomomi Fujisawa, Tatsuya Toyama, Hidetoshi Kawaguchi, Kojiro Mashino, Yuichi Tanino, Satoshi Morita, Masakazu Toi, Shinji Ohno

**Affiliations:** 1 Department of Breast and Endocrine Surgery, Graduate School of Medical Sciences, Kumamoto University, 1-1-1 Honjo, Chuo-ku, Kumamoto, 860-8556 Japan; 2 Department of Breast Surgery, Tenri Yorozu Hospital, Nara, Japan; 3 Department of Surgery, Kurume University School of Medicine, Fukuoka, Japan; 4 Department of Breast Oncology, Aichi Cancer Center Hospital, Aichi, Japan; 5 Department of Breast Surgery, Nagoya City University Graduate School of Medical Sciences, Aichi, Japan; 6 Division of Breast Surgery, Chiba Cancer Center, Chiba, Japan; 7 Department of Breast Surgery, Sagara Hospital, Kagoshima, Japan; 8 Department of Breast Surgery, NHO Hokkaido Cancer Center, Hokkaido, Japan; 9 Department of Breast Surgery, St. Marianna University School of Medicine, Kanagawa, Japan; 10 Department of Breast Surgery, Tokyo Medical University, Tokyo, Japan; 11 Department of Breast Surgery, Osaka International Cancer Institute, Osaka, Japan; 12 Department of Breast Surgery, Hiroshima City Hiroshima Citizens Hospital, Hiroshima, Japan; 13 Department of Medical Oncology, Toranomon Hospital, Tokyo, Japan; 14 Department of Breast Oncology, Gunma Prefectural Cancer Center, Gunma, Japan; 15 Department of Breast Surgery, Matsuyama Red Cross Hospital, Ehime, Japan; 16 Department of Surgery (Breast Surgery), Oita Prefectural Hospital, Oita, Japan; 17 Department of Breast and Endocrine Surgery, Kobe University Hospital, Hyogo, Japan; 18 Department of Biomedical Statistics and Bioinformatics, Kyoto University Graduate School of Medicine, Kyoto, Japan; 19 Department of Breast Surgery, Graduate School of Medicine, Kyoto University, Kyoto, Japan; 20 Breast Oncology Center, Cancer Institute Hospital, Tokyo, Japan

**Keywords:** Bevacizumab, Paclitaxel, Locally advanced breast cancer, Metastatic breast cancer, Overall survival, First line, Second line

## Abstract

**Purpose:**

To investigate the effectiveness and safety of bevacizumab–paclitaxel combination therapy as first- or second-line chemotherapy for HER2-negative locally advanced or metastatic breast cancer in daily clinical practice.

**Methods:**

In this prospective multicenter observational study, bevacizumab–paclitaxel was administered at the discretion of attending physicians. Cohorts A and B had hormone receptor-positive and triple-negative breast cancer (TNBC), respectively. Primary endpoint was overall survival (OS). Multivariate analyses were conducted to identify prognostic factors.

**Results:**

Between November 2012 and October 2014, 767 patients were enrolled from 155 institutions across Japan. Effectiveness was analyzed in 754 eligible patients (cohort A, 539; cohort B, 215) and safety in 750 treated patients (median observation period, 19.7 months). Median OS (95% CI) was 21.7 (19.8–23.6) months in eligible patients; 25.2 (22.4–27.4) months and 13.2 (11.3–16.6) months in cohorts A and B, respectively; and 24.4 (21.9–27.2) months and 17.6 (15.2–20.0) months in patients receiving first- and second-line therapy, respectively. Factors affecting OS (hazard ratio 95% CI) were TNBC (1.75, 1.44–2.14), second-line therapy (1.35, 1.13–1.63), ECOG performance status ≥ 1 (1.28, 1.04–1.57), taxane-based chemotherapy (0.65, 0.49–0.86), cancer-related symptoms (0.56, 0.46–0.68), and visceral metastasis (0.52, 0.40–0.66). Incidences of grade ≥ 3 AEs hypertension, neutropenia, peripheral neuropathy, proteinuria, and bleeding were 35.7%, 27.2%, 7.2%, 3.7%, and 0.3%, respectively.

**Conclusions:**

In Japanese clinical practice, combined bevacizumab–paclitaxel was as effective as in previous studies. Factors that independently predicted poor prognosis in the present study are consistent with those identified previously.

**Trial registration:**

Trial no. UMIN000009086.

**Electronic supplementary material:**

The online version of this article (10.1007/s12282-020-01138-4) contains supplementary material, which is available to authorized users.

## Introduction

Bevacizumab is a humanized monoclonal antibody for vascular endothelial growth factor (VEGF), which is the most important regulator for angiogenesis in both healthy and pathological states [1]. Its enhanced expression is observed in many types of tumors and promotes tumor growth and metastasis [2]. Bevacizumab binds to VEGF, thereby inhibiting VEGF binding to VEGF receptors 1 and 2 on endothelial cells. The consequent inhibition of tumor angiogenesis at the tumor site is understood to suppress the growth of cancer cells [3]. Additionally, normalization of abnormal vessels in the tumor tissue reduces its interstitial pressure, thereby facilitating penetration by anticancer agents in combination with bevacizumab [4].

A meta-analysis on addition of bevacizumab to chemotherapy for patients with locally advanced or metastatic breast cancer (LA/mBC) showed that addition of bevacizumab to first- or second-line chemotherapy significantly prolongs progression-free survival (PFS) and overall response rate (ORR) but not overall survival (OS) [5]. However, another meta-analysis of factors indicating poor prognosis in patients with LA/mBC showed that addition of bevacizumab to first-line chemotherapy improves 1-year OS and OS in patients with poor prognostic factors, as compared with chemotherapy alone [6]. Regarding adverse events (AEs), addition of bevacizumab increases the incidence of hypertension, proteinuria, and bleeding; however, the incidence of thromboembolism or gastrointestinal perforation is unchanged and that of treatment-related deaths is low [5].

The JO19901 study, carried out in Japan, was a phase II study of bevacizumab plus paclitaxel in chemotherapy-naive patients with HER2-negative LA/mBC [7]. The primary efficacy endpoint, median PFS, was 12.9 months. Regarding secondary endpoints, ORR was 74% and median OS was 35.8 months. Regarding safety, no new serious AEs were detected. Thus, the study confirmed the reproducibility in Japanese patients of the efficacy and safety results achieved for bevacizumab plus paclitaxel combination therapy in studies conducted outside Japan.

Although several cohort studies have been carried out in other countries [8–11], clinical experience of bevacizumab plus paclitaxel combination therapy in Japan has been limited to the small number of patients in the JO19901 study, which enrolled 120 patients [7]. Therefore, we conducted a prospective multicenter observational study to investigate the effectiveness and safety of this combination as first- or second-line therapy for LA/mBC in daily clinical practice. Two cohorts, one comprising patients with hormone receptor-positive breast cancer and the other comprising those with triple-negative breast cancer, were established to enable comparison of prognostic factors in patients with each of these cancer subtypes and in patients receiving first- or second-line therapy.

## Patients and methods

### Study design

In this multicenter prospective observational cohort study, patients who met the following inclusion criteria were enrolled: histologically confirmed HER2-negative LA/mBC with confirmed HR status; Eastern Cooperative Oncology Group (ECOG) performance status (PS), 0–3; no history of second-line chemotherapy for LA/mBC; and sufficient bone marrow and major organ functions determined by the attending physician. Exclusion criteria included history of hypersensitivity to the ingredients of bevacizumab or paclitaxel, history of hemoptysis, uncontrolled hypertension, thromboembolism, positive urinary protein test result (≥ 2 +), gastrointestinal perforation, and severe fistula.

Patients were enrolled through central registration and classified by HR status: cohort A comprised patients with HR-positive breast cancer, and cohort B, those with triple-negative breast cancer (TNBC). First-line therapy was defined as treatment for patients who had not previously received chemotherapy for LA/mBC. Second-line therapy was defined as treatment for disease progression after or during receipt of first-line chemotherapy for LA/mBC. In cases of relapse during adjuvant chemotherapy, the first treatment after the relapse was considered the second-line therapy.

Written informed consent was obtained from all patients. The study protocol, procedures, and consent forms were approved by the institutional review board of each participating institution. The study has been registered with the University Hospital Medical Information Network Clinical Trials Registry (https://www.umin.ac.jp/ctr/index-j.htm; trial no. UMIN000009086).

### Study treatment

Because the study was an observational study conducted in a clinical setting, dosage, treatment schedule, and criteria for dose reduction, interruption, and discontinuation were not specified. However, the study protocol recommended the following standard treatment regimen, which was used in the JO19901 study [7]: bevacizumab 10 mg/kg given every 2 weeks, and paclitaxel 90 mg/m^2^ given every week for 3 weeks, followed by a 1-week rest. Each combination of bevacizumab and paclitaxel administered as above for 4 weeks was deemed one cycle.

In cases of discontinuation of either drug due to AEs, the other drug could be continued as monotherapy. The protocol did not specify any treatment after discontinuation.

### Study assessment

At screening on registration, medical history, symptoms of cancer, physical findings, pathological findings relating to the primary and metastatic lesions, presence or absence of measurable lesions, and previous treatments were recorded. During the treatment period, treatment schedule, treatment discontinuations, dose reductions, treatment interruption, concomitant drugs, and the last dosing date were recorded by electronic data capture.

Regarding safety, the incidence of five selected AEs of bevacizumab plus paclitaxel (i.e. neutropenia, hypertension, proteinuria, bleeding, and peripheral neuropathy), of any grade, was recorded. For other AEs, only those of grade ≥ 3 were recorded. AEs were evaluated based on CTCAE version 4.0 (Japanese Clinical Oncology Group edition) [12]. Effectiveness was evaluated and recorded in accordance with the Response Evaluation Criteria in Solid Tumors (RECIST), version 1.1 (Japanese Clinical Oncology Group edition) [13]. At the end of the observational period, patients’ disease progression, death, and post-treatment status were recorded.

### Analysis populations and endpoints

The eligible patient population (used for the effectiveness analysis) was defined as patients who were registered according to the registration procedure, excluding those with ineligible cases or registration error. The treated patient population (used for the safety analysis and the sensitive analysis) was defined as patients who received bevacizumab plus paclitaxel combination therapy at least once. All evaluations were done by attending physicians.

The primary endpoint was OS, defined as the period between date of registration and death from any cause. Secondary endpoints were PFS, ORR, and safety. PFS was defined as the period between the registration date and the day when disease progression was determined (if that occurred first) or death (all causes).

### Statistical analyses

This was an observational study conducted in the setting of daily clinical practice; therefore, the sample size was determined based on feasibility, considering the number of participating institutions, length of the registration period, and epidemiology of patients with HER2-negative LA/mBC. Consequently, the target numbers of patients were determined as 500 for cohort A and 250 for cohort B.

Expected median OS in each cohort according to treatment line (i.e. first- or second-line therapy) was estimated based on data from the prospective studies [7, 8, 14, 15, 17, 18]. Consequently, the expected median OS was 29.0 months and 18.0 months in patients receiving the study treatment as first-line and second-line therapy, respectively, in cohort A, and 17.0 months and 13.0 months in those receiving it as first-line and second-line therapy, respectively, in cohort B. Because the present study included patients who received the study treatment as both first- and second-line therapy, the ratio of first-line therapy patients to second-line therapy patients was assumed to be 5:5–7:3. Therefore, median OS was estimated to be 23.8 months in cohort A patients and 15.2 months in cohort B patients.

For the eligible patient population, cumulative survival curves for OS, median OS, and survival rate in each year were estimated using the Kaplan–Meier method, and Greenwood’s formula was used to construct 95% confidential intervals (CIs). Subgroup analysis was performed by Cox regression analysis to identify important prognostic factors. Sensitivity analysis was also performed, using data from the treated patient population. The same analyses were performed for PFS as those for OS. ORR was calculated as the proportion of patients achieving complete or partial response as the best overall response in patients with measurable lesions. CIs were calculated using the Clopper–Pearson method.

Safety was assessed using data from the treated patient population. The numbers of AEs, their grades, and their causal relation with the study drug were tabulated.

## Results

### Study population and baseline patient characteristics

A total of 767 patients were enrolled from 155 institutions across Japan between November 2012 and October 2014. Patient disposition is shown in Supplementary Fig. 1. Of these, the eligible patient population comprised 754 patients after exclusion of ineligible cases. Within this group, 539 (71.5%) were in cohort A and 215 (28.5%) in cohort B. The numbers of patients receiving the study treatment as first- and second-line therapy were 478 (63.4%) and 276 (36.6%), respectively. The treated patient population, that is, those who received the study treatment at least once, comprised 750 patients.

Baseline characteristics of the eligible patient population are shown in Table 1 and Supplementary Table 1a. Median age was 58 years. Most patients had distant metastasis (86.1%). Of these patients, most had visceral metastasis (91.7%), with ≥ 3 organs affected in a minority of cases (14.0%). Symptoms related to cancer (e.g. pain, dyspnea, pleural effusion, ascites, skin ulcer, and tumor fever) were experienced by 57.6% of eligible patients. Baseline characteristics of the treated patient population are shown in Supplementary Table 1c and are similar to those of the eligible patient population.

**Table 1:** Baseline characteristics (eligible patients)

	All eligible patients		Cohort A^a^		Cohort B^b^		First-line therapy		Second-line therapy	
	*N*	(%)	*n*	(%)	*n*	(%)	*n*	(%)	*n*	(%)
No. of patients	754	100	539	100	215	100	478	100	276	100
Median age (range) (years)	58.0	(26–83)	58.0	(26–81)	58.0	(27–83)	59.0	(26–83)	57.0	(28–83)
Menopausal status										
Premenopausal	198	26.3	133	24.7	65	30.2	127	26.6	71	25.7
Postmenopausal	532	70.6	385	71.4	147	68.4	337	70.5	195	70.7
Unknown	24	3.2	21	3.9	3	1.4	14	2.9	10	3.6
ECOG PS										
0	522	69.2	371	68.8	151	70.2	345	72.2	177	64.1
1	172	22.8	122	22.6	50	23.3	96	20.1	76	27.5
2	43	5.7	34	6.3	9	4.2	29	6.1	14	5.1
3	17	2.3	12	2.2	5	2.3	8	1.7	9	3.3
ER status										
Negative	208	27.6	8	1.5	200	93.0	125	26.2	83	30.1
Positive	544	72.1	529	98.1	15	7.0	351	73.4	193	69.9
Unknown	2	0.3	2	0.4	0	0.0	2	0.4	0	0.0
PgR status										
Negative	332	44.0	122	22.6	210	97.7	203	42.5	129	46.7
Positive	419	55.6	414	76.8	5	2.3	272	56.9	147	53.3
Unknown	3	0.4	3	0.6	0	0.0	3	0.6	0	0.0
Nuclear grade										
1	120	15.9	102	18.9	18	8.4	79	16.5	41	14.9
2	106	14.1	82	15.2	24	11.2	70	14.6	36	13.0
3	216	28.6	111	20.6	105	48.8	144	30.1	72	26.1
Unknown	312	41.4	244	45.3	68	31.6	185	38.7	127	46.0
Ki67 index										
< 30	141	18.7	105	19.5	36	16.7	103	21.5	38	13.8
≥ 30	191	25.3	93	17.3	98	45.6	130	27.2	61	22.1
Unknown	422	56.0	341	63.3	81	37.7	245	51.3	177	64.1
Diagnosis										
Locally advanced	34	4.5	20	3.7	14	6.5	29	6.1	5	1.8
Stage IV	199	26.4	149	27.6	50	23.3	130	27.2	69	25.0
Recurrence	521	69.1	370	68.6	151	70.2	319	66.7	202	73.2
Disease-free interval (months)										
0	233	30.9	169	31.4	64	29.8	159	33.3	74	26.8
0–24	178	23.6	83	15.4	95	44.2	101	21.1	77	27.9
≥ 4	292	38.7	246	45.6	46	21.4	190	39.7	102	37.0
Unknown	51	6.8	41	7.6	10	4.7	28	5.9	23	8.3
Distant metastasis										
No	81	10.7	47	8.7	34	15.8	34	7.1	47	17.0
Yes	649	86.1	476	88.3	173	80.5	422	88.3	227	82.2
Unknown	24	3.2	16	3.0	8	3.7	22	4.6	2	0.7
Metastatic site^c^										
Non-visceral	54	8.3	36	7.6	18	10.4	40	9.5	14	6.2
Visceral	595	91.7	440	92.4	155	89.6	382	90.5	213	93.8
No. of metastatic organs^c^										
< 3	558	86.0	409	85.9	149	86.1	355	84.1	203	89.4
≥ 3	91	14.0	67	14.1	24	13.9	67	15.9	24	10.6
Cancer-related symptoms										
No	315	41.8	230	42.7	85	39.5	200	41.8	115	41.7
Yes	434	57.6	305	56.6	129	60.0	275	57.5	159	57.6
Unknown	5	0.7	4	0.7	1	0.5	3	0.6	2	0.7
Treatment line for locally advanced or metastatic breast cancer										
First line	478	63.4	345	64.0	133	61.9				
Second line	276	36.6	194	36.0	82	38.1				
History of adjuvant therapy^d^										
Chemotherapy	370	71.0	238	64.3	132	87.4	227	71.2	143	70.8
Anthracycline	297	57.0	188	50.8	109	72.2	186	58.3	111	55.0
Taxane	262	50.3	153	41.4	109	72.2	165	51.7	97	48.0
Endocrine therapy	336	64.5	324	87.6	12	7.9	205	64.3	131	64.9
Previous therapy for locally advanced or metastatic breast cancer										
Chemotherapy	266	35.3	188	34.9	78	36.3	12	2.5	254	92.0
Anthracycline	80	10.6	63	11.7	17	7.9	4	0.8	76	27.5
Taxane	54	7.2	36	6.7	18	8.4	5	1.0	49	17.8
Endocrine therapy	356	47.2	348	64.6	8	3.7	195	40.8	161	58.3
Radiotherapy	140	18.6	111	20.6	29	13.5	62	13.0	78	28.3

*ECOG PS* Eastern Cooperative Oncology Group Performance Status, *ER* estrogen receptor, *PgR* progesterone receptor

^a^Patients with hormone receptor-positive breast cancer

^b^Patients with triple-negative breast cancer

^c^Number (%) of distant metastasis

^d^Number (%) of patients with breast cancer recurrence

The proportions of cohort B patients with distant metastasis and metastasis to ≥ 3 organs were generally higher in those receiving first-line therapy than in those receiving second-line therapy; however, there were no differences for the other prognostic factors (Supplementary Table 1a).

### Treatment exposure

Most patients received treatment in accordance with the treatment regimen used in the JO19901 study [7]. Details of treatment exposure in eligible patients are shown in Table 2 and Supplementary Table 2a. Median duration of bevacizumab and paclitaxel exposure was 5.1 and 4.9 months, respectively. Contrary to our expectation, duration of bevacizumab monotherapy after discontinuation of bevacizumab in combination with paclitaxel was extremely short and about 90% of cases discontinued bevacizumab at almost the same time as paclitaxel was discontinued (Table2, Supplementary Table 2a–c).

**Table 2:** Treatment exposure (eligible patients)

	All eligible patients		Cohort A^a^		Cohort B^b^		First-line therapy		Second-line therapy	
	*N*	(%)	*n*	(%)	*n*	(%)	*n*	(%)	*n*	(%)
No. of patients	754	100	539	100	215	100	478	100	276	100
Median duration of study treatment (25th, 75th percentiles), months	5.1	(3.1, 8.7)	5.5	(3.3, 9.5)	4.0	(2.3, 6.5)	5.3	(3.0, 8.7)	4.8	(2.4, 8.8)
Median duration of bevacizumab (25th, 75th percentiles), months	5.1	(2.8, 8.5)	5.4	(3.3, 9.5)	3.7	(2.3, 6.2)	5.1	(2.8, 8.5)	4.6	(2.4, 8.7)
Median RDI of bevacizumab (25th, 75th percentiles),	97.5	(86.2, 100)	95.9	(86.5, 100)	100	(85.7, 100)	95.6	(87.2–100)	100	(84.8–100)
Discontinuations of bevacizumab, *n* (%)	748	99.2	536	99.4	212	98.6	475	99.4	273	98.9
Reason for discontinuation of bevacizumab, *n* (%)										
Disease progression	379	50.7	261	48.7	118	55.7	221	46.5	158	57.9
Adverse events	214	28.6	169	31.5	45	21.2	143	30.1	71	26.0
Other	152	20.3	105	19.6	47	22.2	109	22.9	43	15.8
Unknown	3	0.4	1	0.2	2	0.9	2	0.4	1	0.4
Bevacizumab dose reductions, *n* (T)	15	2.0	12	2.2	3	1.4	13	2.7	2	0.7
Reason for bevacizumab dose reduction, *n* (%)^c^										
Hypertension	3	20.0	2	16.7	1	33.3	3	23.1	0	0.0
Proteinuria	3	20.0	3	25.0	0	0.0	3	23.1	0	0.0
Bleeding	1	6.7	1	8.3	0	0.0	1	7.7	0	0.0
Neutropenia	1	6.7	1	8.3	0	0.0	0	0.0	1	50.0
Other adverse events	3	20.0	3	25.0	0	0.0	3	23.1	0	0.0
Other	4	26.7	2	16.7	2	66.7	3	23.1	0	0.0
Bevacizumab dose interruptions or delays, *n* (%)	263	34.9	188	34.9	75	34.9	155	32.4	108	39.1
Reason for bevacizumab dose interruption or delay, *n* (%)^c^										
Hypertension	18	6.8	15	8.0	3	4.0	16	10.3	2	1.9
Proteinuria	53	20.2	36	19.1	17	22.7	35	22.6	18	16.7
Bleeding	3	1.1	2	1.1	1	1.3	2	1.3	1	0.9
Neutropenia	77	29.3	61	32.4	16	21.3	38	24.5	39	36.1
Other adverse events	106	40.3	77	41.0	29	38.7	61	39.4	45	41.7
Other	114	43.3	79	42.0	35	46.7	63	40.6	51	47.2
Median duration of paclitaxel (25th, 75th percentiles), months	4.9	(2.8, 8.1)	5.3	(3.2, 9.0)	3.9	(2.3, 6.0)	5.1	(3.0, 8.1)	4.6	(2.4, 8.2)
Median RDI of paclitaxel (25th, 75th percentiles),	90.9	(70.6, 100)	88.9	(69.5, 100)	96.0	(75.6, 105)	91.7	(72.7, 100)	89.8	(68.6, 100)
Discontinuations of paclitaxel, *n* (%)	748	99.2	536	99.4	212	98.6	475	99.4	273	98.9
Reason for discontinuation of paclitaxel, *n* (%)										
Disease progression	363	48.5	245	45.7	118	55.7	216	45.5	147	53.8
Adverse events	246	32.9	198	36.9	48	22.6	158	33.3	88	32.2
Other	136	18.2	92	17.2	44	20.8	99	20.8	37	13.6
Unknown	3	0.4	1	0.2	2	0.9	2	0.4	1	0.4
Paclitaxel dose reductions, *n* (%)	276	36.6	208	38.6	68	31.6	185	38.7	91	33.0
Reason for paclitaxel dose reduction, *n* (%)^c^										
Peripheral neutropenia	114	41.3	89	42.8	25	36.8	81	43.8	33	36.3
Neutropenia	117	42.4	88	42.3	29	42.6	67	36.2	50	54.9
Other adverse events	81	29.3	61	29.3	20	29.4	57	30.8	24	26.4
Other	28	10.1	20	9.6	8	11.8	19	10.3	9	9.9
Paclitaxel dose interruptions or delays, *n* ()	351	46.6	259	48.1	92	42.8	207	43.3	144	52.2
Reason for paclitaxel dose interruption or delay, *n* (%)^c^										
Peripheral neutropenia	65	18.5	52	20.1	13	14.1	39	18.8	26	18.1
Neutropenia	159	45.3	123	47.5	36	39.1	82	39.6	77	53.5
Other adverse events	168	47.9	118	45.6	50	54.3	94	45.4	74	51.4
Other	127	36.2	90	34.7	37	40.2	72	34.8	55	38.2
Median duration of bevacizumab monotherapy after discontinuation of bevacizumab + paclitaxel (25 percentile, 75 percentile), months	1.4 (*N* = 79)	0.5, 3.9	1.4 (*N* = 65)	0.5, 3.9	0.6 (*N* = 14)	0.2, 3.0	1.4 (*N* = 52)	0.5, 3.9	0.8 (*N* = 27)	0.3, 3.7
Median duration of paclitaxel monotherapy after discontinuation of bevacizumab + paclitaxel (25 percentile, 75 percentile), months	0.2 (*N* = 133)	0.2, 0.7	0.2 (*N* = 86)	0.2, 0.7	0.2 (*N* = 47)	0.2, 0.9	0.2 (*N* = 87)	0.2, 1.2	0.2 (*N* = 46)	0.2, 0.3

*RDI* relative dose intensity

^a^Patients with hormone receptor-positive breast cancer

^b^Patients with triple-negative breast cancer

^c^Multiple items could be selected

Of the 754 eligible patients, 748 (99.2%) discontinued the study treatment; of these, 28.6% and 32.9% discontinued bevacizumab and paclitaxel, respectively, due to AEs. Regarding discontinuations due to other reasons, those recorded for ≥ 1% of patients included patient request (4.9%), maximum response (2.8%), breast surgery (2.7%), completion of scheduled treatment (2.3%), and treatment for other disease (1.5%).

The dose of bevacizumab or paclitaxel was reduced due to AEs in 1.5% and 33.1%, respectively, and it was suspended due to AEs in 19.7% and 29.7%, respectively.

When the treatment schedule of bevacizumab plus paclitaxel was the same as that in the JO19901 study [7], relative dose intensity of bevacizumab and paclitaxel was 99.2% and 90.9%, respectively.

Details of treatment exposure for patents in the treated patient population are shown in Supplementary Table 2b, c. Treatment exposure in this population was similar to that in the eligible patient population.

### Effectiveness

#### Overall survival

**Fig. 1:**
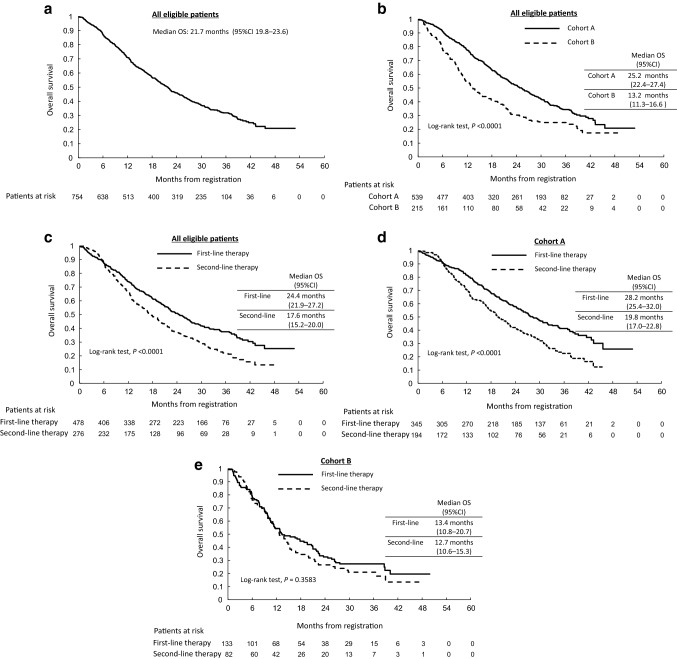
Overall survival in the eligible patient population: **a** all eligible patients; **b** cohort A (patients with hormone receptor-positive breast cancer) versus cohort B (patients with triple-negative breast cancer); **c**, all eligible patients receiving first-line versus second-line therapy; **d** first-line versus second-line therapy in cohort A; **e** first-line versus second-line therapy in cohort B

Median observation period was 19.7 months. Events occurred in 496 of the 754 eligible patients (65.8%) during observation. Median OS was 21.7 months (95% CI 19.8–23.6 months), 25.2 months (95% CI 22.4–27.4 months), 13.2 months (95% CI 11.3–16.6 months), 24.4 months (95% CI 21.9–27.2 months), and 17.6 months (95% CI 15.2–20.0 months) in the full eligible patient population, in cohort A, in cohort B, in patients receiving the study treatment as first-line chemotherapy, and in those receiving it as second-line chemotherapy, respectively (Fig. 1a–c). Additionally, 1-year OS was 71.0%, 77.6%, 54.3%, 74.1%, 65.7%, in the full eligible patient population, in cohort A, in cohort B, in patients receiving the study treatment as first-line chemotherapy, and in those receiving it as second-line chemotherapy, respectively. Details of OS for eligible populations by cohort and treatment-line are shown in Supplementary Table 3 and Fig. 1d, e. Interestingly, OS was significantly longer in patients receiving the study treatment as first-line therapy than in those receiving it as second-line therapy in cohort A (log-rank test *p* < 0.0001, Fig. 2d), but not in cohort B (*p* = 0.3583, Fig. 1e).

The results of multivariate analysis for OS in the eligible patient population are summarized in Table 3a. In decreasing order of hazard ratio (HR), the baseline characteristics independently associated with OS were TNBC, second-line therapy, ECOG PS ≥ 1, neoadjuvant or adjuvant taxane-based chemotherapy, cancer-related symptoms, and visceral metastasis.

**Table 3:** Results of univariate and multivariate analyses for overall survival

(a) All eligible patients							
	Univariate analysis (*N* = 754)				Multivariate analysis (*N* = 736)^a^		
Variable	*N*	HR	95% CI	*p*	HR	95% CI	*p*
Cohort A vs cohort B	754	1.63	1.35–1.97	< 0.0001	1.75	1.44–2.14	< 0.0001
First- vs second-line therapy	754	1.46	1.22–1.74	< 0.0001	1.35	1.13–1.63	0.0011
Age: < 50 years vs ≥ 50 years	754	0.92	0.77–1.10	0.3361			
ECOG PS: 0 vs 1, 2, or 3	754	1.59	1.32–1.91	< 0.0001	1.28	1.04–1.57	0.0175
Visceral metastasis: yes vs no	754	0.55	0.44–0.70	< 0.0001	0.52	0.40–0.66	< 0.0001
Cancer-related symptoms: yes vs no	749	0.57	0.48–0.69	< 0.0001	0.56	0.46–0.68	< 0.0001
Neoadjuvant or adjuvant chemotherapy: yes vs no	741	0.63	0.53–0.75	< 0.0001	0.87	0.66–1.14	0.3139
Neoadjuvant or adjuvant taxane-based chemotherapy: yes vs no	741	0.60	0.50–0.72	< 0.0001	0.65	0.49–0.86	0.0026
History of taxane-based chemotherapy: yes vs no	754	0.90	0.64–1.26	0.5380			
History of anthracycline-based chemotherapy: yes vs no	754	1.04	0.78–1.38	0.8108			
History of hormone therapy: yes vs no	754	1.02	0.86–1.22	0.8031			
Nuclear grade: ≤ 2 vs 3	442	1.31	1.05–1.64	0.0186			
Ki-67 index: < 30 vs ≥ 30	332	1.64	1.25–2.16	0.0004			
Disease-free interval: 0 (advanced breast cancer) vs ≤ 24 months vs > 24 months	703	1.02	0.92–1.13	0.7575			

(b) Patients with recurrent breast cancer							
	Univariate analysis (*n* = 521)				Multivariate analysis (*n* = 456)^a^		
Variable	*n*	HR	95% CI	*P*	HR	95% CI	*P*
Cohort A vs cohort B	521	1.52	1.22–1.90	0.0002	1.27	0.94–1.71	0.1251
First- vs second-line therapy	521	1.33	1.08–1.64	0.0069	1.20	0.95–1.52	0.1210
Age: < 50 years vs ≥ 50 years	521	0.92	0.75–1.13	0.4072			
ECOG PS: 0 vs 1, 2, or 3	521	1.65	1.32–2.06	< 0.0001	1.32	1.02–1.71	0.0333
Visceral metastasis: yes vs no	521	0.58	0.44–0.77	0.0001	0.53	0.39–0.72	0.0001
Cancer-related symptoms: yes vs no	518	0.50	0.41–0.62	< 0.0001	0.52	0.41–0.66	< 0.0001
Neoadjuvant or adjuvant chemotherapy: yes vs no	508	0.68	0.53–0.86	0.0016	1.08	0.76–1.54	0.6634
Neoadjuvant or adjuvant taxane-based chemotherapy: yes vs no	508	0.66	0.53–0.81	0.0001	0.70	0.51–0.95	0.0209
History of taxane-based chemotherapy: yes vs no	521	0.86	0.56–1.32	0.4944			
History of anthracycline-based chemotherapy: yes vs no	521	0.88	0.57–1.36	0.5617			
History of hormone therapy: yes vs no	521	1.28	1.04–1.57	0.0190	1.14	0.87–1.50	0.3367
Nuclear grade: ≤ 2 vs 3	299	1.17	0.90–1.52	0.2389			
Ki-67 index: < 30 vs ≥ 30	178	1.81	1.27–2.57	0.0011			
Disease-free interval 1: ≤ 24 months vs > 24 months	470	0.45	0.36–0.56	< 0.0001	0.50	0.39–0.63	< 0.0001

*CI* confidence interval, *ECOG PS* Eastern Cooperative Oncology Group Performance Status, *HR* hazard ratio

Variables with a significance level < 0.15 in the univariate analysis and without ≥ 0.67 missing values were included in the multivariate analysis

The results of multivariate analysis in the 521 patients with recurrent breast cancer are summarized in Table 3b. The following baseline characteristics were identified as independent predictors of OS: ECOG PS ≥ 1, neoadjuvant or adjuvant taxane-based chemotherapy, visceral metastasis, cancer-related symptoms, and disease-free interval (DFI) ≤ 24 months.

The results of univariate and multivariate analyses of baseline characteristics associated with OS by cohort are shown in Supplementary Tables 4 and 5.

#### Progression-free survival and objective response rates

Median PFS was 8.5 months (95% CI 7.8–9.2 months), 9.4 months (95% CI 8.7–10.7 months), 6.0 months (95% CI 5.5–7.4 months), 9.3 months (95% CI 8.5–10.7 months), and 7.2 months (95% CI 6.0–8.4 months) in the full eligible patient population, in cohort A, in cohort B, in patients receiving the study treatment as first-line chemotherapy, and in those receiving it as second-line chemotherapy, respectively (Supplementary Fig. 2a, 2b, 2c). Details of PFS for eligible populations by cohort and treatment line are shown in Supplementary Table 6 and Supplementary Fig. 2d, 2e.

Multivariate analysis identified the following baseline characteristics independently associated with PFS (Table 4a): TNBC, ECOG PS ≥ 1, history of endocrine therapy, cancer-related symptoms, history of neoadjuvant or adjuvant chemotherapy, history of neoadjuvant or adjuvant taxane-based chemotherapy, and visceral metastasis.

**Table 4:** Results of univariate and multivariate analyses for progression-free survival

(a) All eligible patients							
	Univariate analysis (*n* = 754)				Multivariate analysis (*n* = 687)^a^		
Variable	*n*	HR	95% CI	*P*	HR	95% CI	*P*
Cohort A vs cohort B	754	1.33	1.13–1.58	0.0008	1.56	1.26–1.93	0.0001
First- vs second-line therapy	754	1.40	1.20–1.64	< 0.0001	1.19	0.99–1.42	0.0622
Age: < 50 years vs ≥ 50 years	754	1.00	0.86–1.16	0.9589			
ECOG PS: 0 vs 1, 2, or 3	754	1.60	1.36–1.88	< 0.0001	1.36	1.13–1.64	0.0013
Visceral metastasis: yes vs no	754	0.63	0.52–0.76	< 0.0001	0.68	0.55–0.85	0.0005
Cancer-related symptoms: yes vs no	749	0.68	0.58–0.79	< 0.0001	0.72	0.60–0.86	0.0003
Neoadjuvant or adjuvant chemotherapy: yes vs no	741	0.62	0.53–0.72	< 0.0001	0.71	0.53–0.95	0.0227
Neoadjuvant or adjuvant taxane-based chemotherapy: yes vs no	741	0.61	0.52–0.72	< 0.0001	0.69	0.54–0.89	0.0044
History of taxane-based chemotherapy: yes vs no	754	0.69	0.52–0.92	0.0113	0.82	0.58–1.14	0.2358
History of anthracycline-based chemotherapy: yes vs no	754	1.04	0.80–1.31	0.8458			
History of endocrine therapy: yes vs no	754	0.85	0.73–0.99	0.0411	0.76	0.62–0.93	0.0075
Nuclear grade: ≤ 2 vs 3	442	1.18	0.97–1.44	0.0961			
Ki-67 index: < 30 vs ≥ 30	332	1.54	1.22–1.96	0.0003			
Disease-free interval 1: 0 (advanced breast cancer) vs ≤ 24 months vs > 24 months	703	1.07	0.99–1.17	0.1040	0.89	0.78–1.01	0.0663

(b) Patients with recurrent breast cancer							
	Univariate analysis (*n* = 521)				Multivariate analysis (*n* = 456)^a^		
Variable	*n*	HR	95% CI	*p*	HR	95% CI	*p*
Cohort A vs cohort B	521	1.31	1.08–1.60	0.0073	1.12	0.89–1.40	0.3505
First- vs second-line therapy	521	1.33	1.11–1.60	0.0021	1.16	0.94–1.43	0.1643
Age: < 50 years vs ≥ 50 years	521	1.01	0.84–1.20	0.9470			
PS: 0 vs 1, 2, or 3	521	1.74	1.43–2.11	< 0.0001	1.49	1.19–1.88	0.0006
Visceral metastasis: yes vs no	521	0.74	0.59–0.93	0.0086	0.78	0.60–1.00	0.0518
Cancer-related symptoms: yes vs no	518	0.62	0.52–0.74	< 0.0001	0.67	0.55–0.83	0.0002
Neoadjuvant or adjuvant chemotherapy: yes vs no	508	0.66	0.54–0.81	0.0001	0.85	0.63–1.14	0.2795
Neoadjuvant or adjuvant taxane-based chemotherapy: yes vs no	508	0.67	0.56–0.81	< 0.0001	0.78	0.60–1.01	0.0573
History of taxane-based chemotherapy: yes vs no	521	0.66	0.45–0.97	0.0361	0.75	0.47–1.21	0.2333
History of anthracycline-based chemotherapy: yes vs no	521	0.88	0.60–1.28	0.5067			
History of hormone therapy: yes vs no	521	1.07	0.89–1.28	0.4891			
Nuclear grade: ≤ 2 vs 3	299	1.05	0.83–1.33	0.6669			
Ki-67 index: < 30 vs ≥ 30	178	1.46	1.07–2.00	0.0174			
Disease-free interval 1: ≤ 24 months vs > 24 months	470	0.51	0.42–0.61	< 0.0001	0.54	0.44–0.67	< 0.0001

Variables with a significance level < 0.15 in the univariate analysis and without ≥ 0.67 missing values were included in the multivariate analysis

*CI* confidence interval, *ECOG PS* Eastern Cooperative Oncology Group Performance Status, *HR* hazard ratio

Multivariate analysis also identified several baseline characteristics as independent predictors of prognosis in the 521 patients with recurrent breast cancer (Table 4b): ECOG PS ≥ 1, cancer-related symptoms, and DFI ≤ 24 months.

In the sensitivity analysis, the results for OS and PFS in treated patients were similar to those for the eligible patient population (Supplementary Tables 3 and 6).

ORR in patients with measurable lesions was 56.1%, 59.3%, 48.8%, 62.2%, and 45.1% in the full eligible patient population, in cohort A, in cohort B, in patients receiving the study treatment as first-line chemotherapy, and in those receiving it as second-line chemotherapy, respectively (Table 5a). ORRs by cohort and treatment line are summarized in Table 5b.

**Table 5:** Overall response rate in patients with measurable lesions

(a) All eligible patients							
	All eligible patients	Cohort A^a^	Cohort B^b^	*P*	First-line therapy	Second-line therapy	*p*
No. of patients with target lesions	545	383	162		352	193	
Best response, *n* (%)							
CR	14 (2.6%)	8 (2.1%)	6 (3.7%)	0.0180 (W)	10 (2.8%)	4 (2.1%)	0.0001 (W)
PR	292 (53.6%)	219 (57.2%)	73 (45.1%)		209 (59.4%)	83 (43.0%)	
SD	139 (25.5%)	104 (27.2%)	35 (21.6%)		77 (21.9%)	62 (32.1%)	
PD	71 (13.0%)	36 (9.4%)	35 (21.6%)		35 (9.9%)	36 (18.7%)	
NE	29 (5.3%)	16 (4.2%)	13 (8.0%)		21 (6.0%)	8 (4.1%)	
Response rate, *n* (%)							
CR plus PR	306 (56.1%)	227 (59.3%)	79 (48.8%)	0.0297 (F)	219 (62.2%)	87 (45.1%)	0.0001 (F)
95% CI	51.9–60.4	54.2–64.2	40.8–56.7		56.9–67.3	37.9–52.4	

(b) Cohorts A and B								
	Cohort A^a^	First-line therapy	Second-line therapy	*P*	Cohort B^b^	First-line therapy	Second-line therapy	*p*
No. of patients with target lesions	383	252	131		162	100	62	
Best response								
CR	8 (2.1%)	6 (2.4%)	2 (1.5%)	0.0048 (W)	6 (3.7%)	4 (4.0%)	2 (3.2%)	0.0011 (W)
PR	219 (57.2%)	155 (61.5%)	64 (48.9%)		73 (45.1%)	54 (54.0%)	19 (30.6%)	
SD	104 (27.2%)	57 (22.6%)	47 (35.9%)		35 (21.6%)	20 (20.0%)	15 (24.2%)	
PD	36 (9.4%)	21 (8.3%)	15 (11.5%)		35 (21.6%)	14 (14.0%)	21 (33.9%)	
NE	16 (4.2%)	13 (5.2%)	3 (2.3%)		13 (8.0%)	8 (8.0%)	5 (8.1%)	
Response rate								
CR plus PR	227 (59.3%)	161 (63.9%)	66 (50.4%)	0.0119 (F)	79 (48.8%)	58 (58.0%)	21 (33.9%)	0.0036 (F)
95% CI	54.2–64.2	57.6–69.8	41.5–59.2		40.8–56.7	47.7–67.8	22.3–47.0	

*CR* complete response, *F* Fisher’s exact test, *NE* not evaluable, *PD* progressive disease, *PR* partial response, *SD* stable disease, *W* Wilcoxon rank sum test

^a^Patients with hormone receptor-positive breast cancer

^b^Patients with triple-negative breast cancer

### Safety

The great majority of eligible patients (96.3%) experienced at least one AE, and 63.1% experienced one or more grade ≥ 3 AEs. Table 6 lists the AEs in treated patients. Incidences of grade ≥ 3 AEs hypertension, neutropenia, peripheral neuropathy, proteinuria, and bleeding were 35.7%, 27.2%, 7.2%, 3.7%, and 0.3%, respectively.

**Table 6:** Incidence of adverse events (AEs)

	Treated patient population		Cohort A^a^		Cohort B^b^		First-line therapy		Second-line therapy	
	*n*	(%)	*n*	(%)	*n*	(%)	*n*	(%)	*n*	(%)
(a) Selected AEs										
No. of patients	750	100	538	100	212	100	475	100	275	100
Hypertension										
All grades	602	80.3	429	79.7	173	81.6	383	80.6	219	79.6
Grade ≥ 3	268	35.7	190	35.3	78	36.8	167	35.2	101	36.7
Peripheral neuropathy										
All grades	535	71.3	400	74.3	135	63.7	341	71.8	194	70.5
Grade ≥ 3	54	7.2	41	7.6	13	6.1	36	7.6	18	6.5
Neutropenia										
All grades	347	46.3	256	47.6	91	42.9	203	42.7	144	52.4
Grade ≥ 3	204	27.2	151	28.1	53	25.0	115	24.2	89	32.4
Proteinuria										
All grades	223	29.7	160	29.7	63	29.7	156	32.8	67	24.4
Grade ≥ 3	28	3.7	18	3.3	10	4.7	20	4.2	8	2.9
Bleeding										
All grades	131	17.5	96	17.8	35	16.5	87	18.3	44	16.0
Grade ≥ 3	2	0.3	2	0.4	0	0.0	1	0.2	1	0.4
(b) Bevacizumab-specific AEs other than the selected adverse events (grade ≥ 3)										
No. of patients	750	100	538	100	212	100	475	100	275	100
Congestive heart failure	5	0.7	4	0.7	1	0.5	3	0.6	2	0.7
Gastrointestinal perforation	2	0.3	2	0.4	0	0.0	2	0.4	0	0.0
Thromboembolism	3	0.4	3	0.6	0	0.0	2	0.4	1	0.4
Wound dehiscence	2	0.3	1	0.2	1	0.5	0	0.0	2	0.7
(c) Other adverse events grade ≥ 3										
No. of patients	750	100	538	100	212	100	475	100	275	100
Fatigue	12	1.6	6	1.1	6	2.8	4	0.8	8	2.9
Stomatitis	6	0.8	6	1.1	0	0.0	3	0.6	3	1.1
Febrile neutropenia	4	0.5	2	0.4	2	0.9	1	0.2	3	1.1
Other infections	23	3.1	18	3.3	5	2.4	15	3.2	8	2.9
Skin disorders	11	1.5	10	1.9	1	0.5	9	1.9	2	0.7
Anemia	10	1.3	8	1.5	2	0.9	5	1.1	5	1.8
AST/ALT elevation	10	1.3	6	1.1	4	1.9	8	1.7	2	0.7
Appetite loss	7	0.9	5	0.9	2	0.9	3	0.6	4	1.5
Diarrhea	5	0.7	3	0.6	2	0.9	5	1.1	0	0.0
Drug-induced pneumonitis	5	0.7	4	0.7	1	0.5	2	0.4	3	1.1
Pain	4	0.5	3	0.6	1	0.5	2	0.4	2	0.7
Others	31	4.1	24	4.5	7	3.3	13	2.7	18	6.5

*ALT* alanine aminotransferase, *AST* aspartate aminotransferase

^a^Patients with hormone receptor-positive breast cancer

^b^Patients with triple-negative breast cancer

Serious AEs were recorded in 66 patients (8.8%) including 15 patients with infection, five patients with congestive heart failure and 4 patients with drug-induced pneumonitis, fracture, gastrointestinal perforation, or liver dysfunction (Supplementary Table 7). Treatment-related deaths occurred in 6 patients (0.8%); the causes were liver failure (3 patients), acute gastroenteritis and heart failure (1 patient), gastrointestinal bleeding (1 patient), and gastrointestinal perforation (1 patient). Of the 3 deaths due to liver failure, one had liver failure associated with disease progression, and the other two had so-called pseudocirrhosis, which is associated with liver atrophy due to acute tumor response by chemotherapy on massive liver metastases and disorder of subsequent liver regeneration process.

## Discussion

The B-SHARE study was a prospective observational study to investigate the effectiveness and safety of bevacizumab combined with paclitaxel as first- or second-line chemotherapy for HER2-negative LA/mBC under real-world clinical conditions in Japan. During the median observation period of 19.7 months, median OS for eligible patients was 21.7 months, and median OS for eligible patients receiving first-line therapy was 24.4 months. These results are within the range (21.6–30.2 months) achieved in previous phase III studies [14–16] and observational studies [8–11, 17]. Although there have been no previous observational studies on bevacizumab plus paclitaxel as second-line therapy, median OS for eligible patients receiving second-line therapy in the present study (17.6 months) was similar to the 18.0 months achieved in the RIBBON-2 study conducted as second-line chemotherapy [18], in which the efficacy and safety of bevacizumab combined with standard chemotherapy was compared with standard chemotherapy alone.

The 74.1% 1-year median OS for first-line therapy was a good result and similar to that determined by a meta-analysis of data from randomized controlled studies of bevacizumab combined with chemotherapy as first-line therapy (i.e. 71%) [6], showing that bevacizumab combined with chemotherapy may improve 1-year OS when compared with chemotherapy alone in high-risk patients.

The multivariate analysis results for OS in eligible patients identified TNBC, second-line therapy, poor PS, perioperative history of taxane therapy, cancer-related symptoms, DFI ≤ 2 years (i.e. recurrent breast cancer), and visceral metastasis as independent factors for poor prognosis. This is similar to the findings of previous studies on chemotherapy with [19] or without bevacizumab [20–23].

OS was significantly longer in patients receiving first-line therapy than in those receiving second-line therapy in cohort A but not in cohort B. Regarding baseline characteristics in cohort B, the proportions of patients with distant metastasis and metastasis to ≥ 3 organs were higher in those receiving first-line therapy than in those receiving second-line therapy, but no differences were found for the other factors. After completion of the study treatment, a greater proportion of patients receiving first-line therapy in cohort B were transferred to best supportive care compared with those in cohort A (33.1% and 21.2%, respectively). These findings suggest that patients with TNBC are less likely than those with hormone receptor-positive cancer to continue therapy because of many poor prognostic factors, but when patients were able to undergo second-line and subsequent therapy, they are likely to have a better prognosis.

As for first-line therapy, median PFS in eligible patients was 9.3 months and ORR in those with measurable lesions was 62.2%. As with OS, the results were consistent with those of previous randomized controlled studies [14, 15, 24, 25] and observational studies [8–11, 17]. For second-line therapy, median PFS was 7.2 months and ORR was 45.1%, similar to the results of the RIBBON-2 study [18].

The multivariate analysis results for PFS in eligible patients, including those with advanced disease, identified TNBC, poor PS, history of endocrine therapy, cancer-related symptoms, history of perioperative chemotherapy, history of perioperative taxane, and visceral metastasis as factors indicating poor prognosis. However, in patients with recurrent breast cancer, poor PS, cancer-related symptoms, and DFI ≤ 2 years were independent factors for poor prognosis. Therefore, poor prognostic factors for PFS differed with patient background. Although poor PS and cancer-related symptoms may be considered mutually associated, they were independent poor prognostic factors for both OS and PFS, regardless of whether the cancer was advanced or recurrent. The possibility that cancer-related symptoms are a poor prognostic factor in LA/mBC is supported by several other studies [23, 26, 27].

Despite the similarity in effectiveness (i.e. OS, PFS, and ORR) shown in the present study to that obtained in randomized controlled studies [14–16, 24] and observational studies [8–11, 17], the dosing period for bevacizumab in first-line therapy (5.3 months) was shorter than in randomized controlled studies [24, 25]. In fact, the bevacizumab dosing period tends to be shorter in observational studies [8–11, 17] than in randomized controlled studies [24, 25]. However, the bevacizumab dosing period in a retrospective cohort study [11] using information from the French Epidemiological Strategy and Medical Economics database was similar to that of the present study. The shorter dosing period in the present study compared with in randomized controlled studies may have been due to differences in patient selection (with poorer PS) and adherence to treatment. In the present study, 15.3% of patients were aged ≥ 70 years, and 8.0% had PS of ≥ 2. About 30% of patients discontinued treatment because of AEs, which is similar to that in the randomized controlled studies, whereas about 20% discontinued treatment without having disease progress (e.g. undergoing surgery after tumor shrinkage or switching to endocrine therapy).

No new AEs related to bevacizumab plus paclitaxel were detected in the present study. Incidence of all grades of AEs (96.3%) and those of grade ≥ 3 (63.1%) were higher than in previous randomized controlled studies [14, 18, 24, 25] and observational studies [8–10, 17]. However, there was no increase in the incidence of serious AEs or treatment-related deaths. We experienced 2 cases of treatment-related death due to so-called pseudocirrhosis during treatment of bevacizumab plus paclitaxel. Pseudocirrhosis is characterized by morphological changes in the liver that resembling cirrhosis on the radiological findings without typical histopathology of cirrhosis [28]. Pseudocirrhosis as adverse events by chemotherapy is not rare and an important complication of chemotherapy in patients with liver metastases. Recently, Oliai et al. [29] reported that pseudocirrhosis developed in 37 (55%) of 67 metastatic breast cancer patients with liver metastasis and was associated with poor prognosis in patients with live metastasis. They also described that chemotherapy agents associated with the development of pseudocirrhosis were albumin-bound paclitaxel, capecitabine, cisplatin, everolimus and vinorelbine. This adverse event is not bevacizumab-specific. However, the possibility that bevacizumab may inhibit the process of liver regeneration after treatment-induced hepatic injury cannot be ruled out.

The present study had several limitations. First, it was a single-arm observational study of bevacizumab plus paclitaxel combination therapy, so there was no direct comparison in terms of the effectiveness and safety between bevacizumab plus paclitaxel and paclitaxel alone. Second, treatment effectiveness (PFS and ORR) was assessed by attending physicians, and HR and HER2 status were also assessed at each facility. Central assessment or review was not done for the evaluation of effectiveness and those receptors status. Third, most patients received treatment in accordance with the treatment regimen used in the JO19901 study. Therefore, we could not examine the relationship between the dosage or the schedule of bevacizumab plus paclitaxel and its effectiveness to find the optimal use of this combination. Fourth, because the present study was done under daily clinical conditions, discontinuation due to the wishes of the patient or the decision of the attending physician was possible, regardless of whether the effects of treatment were sustained. During the course of treatment, various strategies were adopted after tumor reduction due to study treatment, such as discontinuation of treatment, switching to hormonal therapy for maintenance, or surgical intervention, which are uncommon in randomized controlled studies. The limitations of the present study make it difficult to obtain a true result for PFS and ORR. However, OS is a robust endpoint and we consider the OS reported here to be close to its true value, because it was achieved in patients treated with bevacizumab plus paclitaxel under real clinical conditions.

In conclusion, bevacizumab plus paclitaxel as first- or second-line chemotherapy in Japanese patients with HER2-negative LA/mBC was as effective as in previous randomized controlled studies and prospective observational studies. Furthermore, the good tolerability of this regimen was confirmed.

## Electronic supplementary material

Below is the link to the electronic supplementary material.

Supplementary file1 (XLSX 85 kb)

Supplementary file2 (PDF 953 kb)
