# Supplementary material for: Prospective observational study of bevacizumab combined with paclitaxel as first- or second-line chemotherapy for locally advanced or metastatic breast cancer: the JBCRG-C05 (B-SHARE) study
Source: Breast Cancer. 2020 Jul 26;28(1):145–60. doi: 10.1007/s12282-020-01138-4 (PMC7796874; doi:10.1007/s12282-020-01138-4)
Supplement: Supplementary file 2 — Supplementary file2 (PDF 953 kb) [file 12282_2020_1138_MOESM2_ESM.pdf]

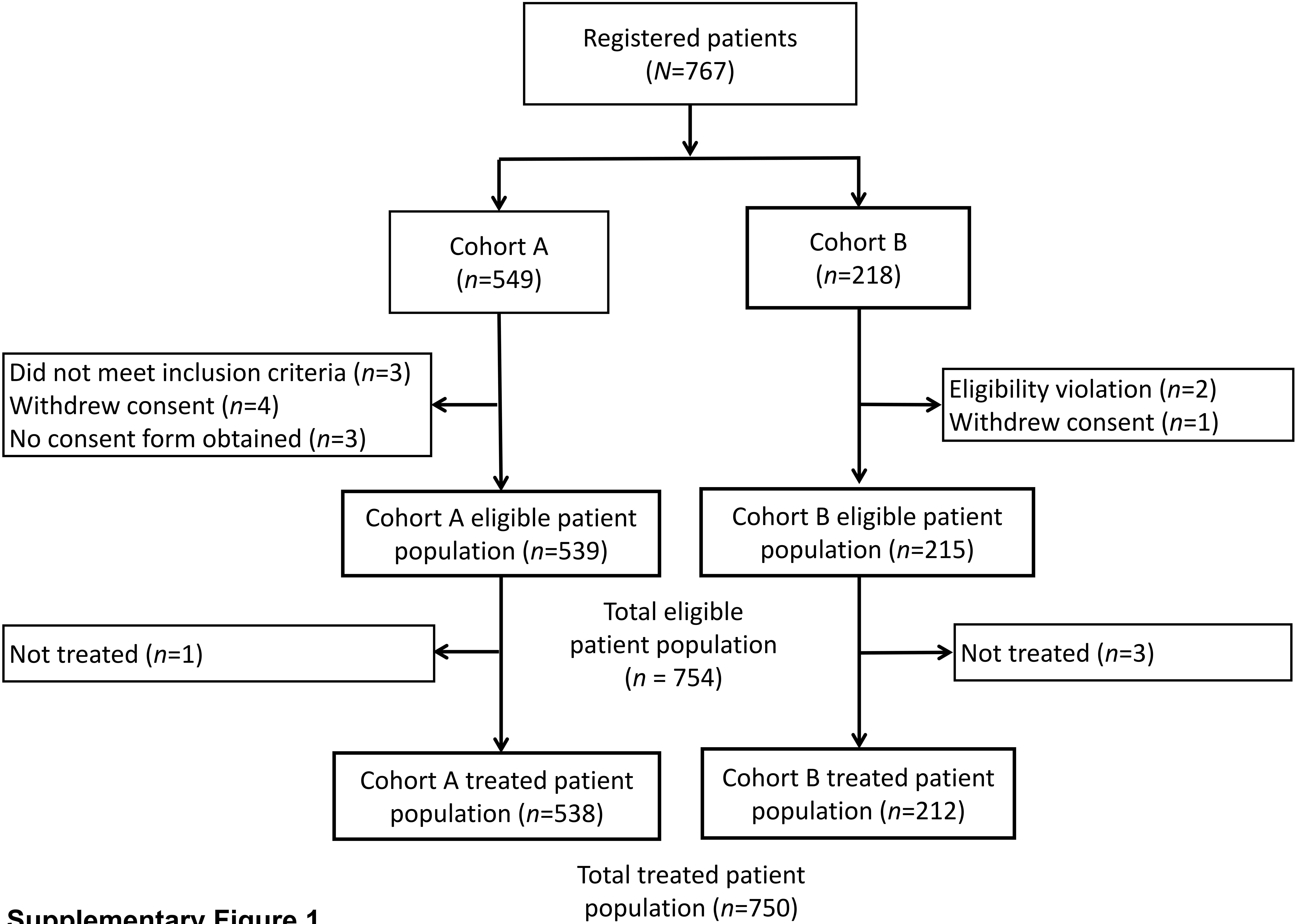

Supplementary Figure 1

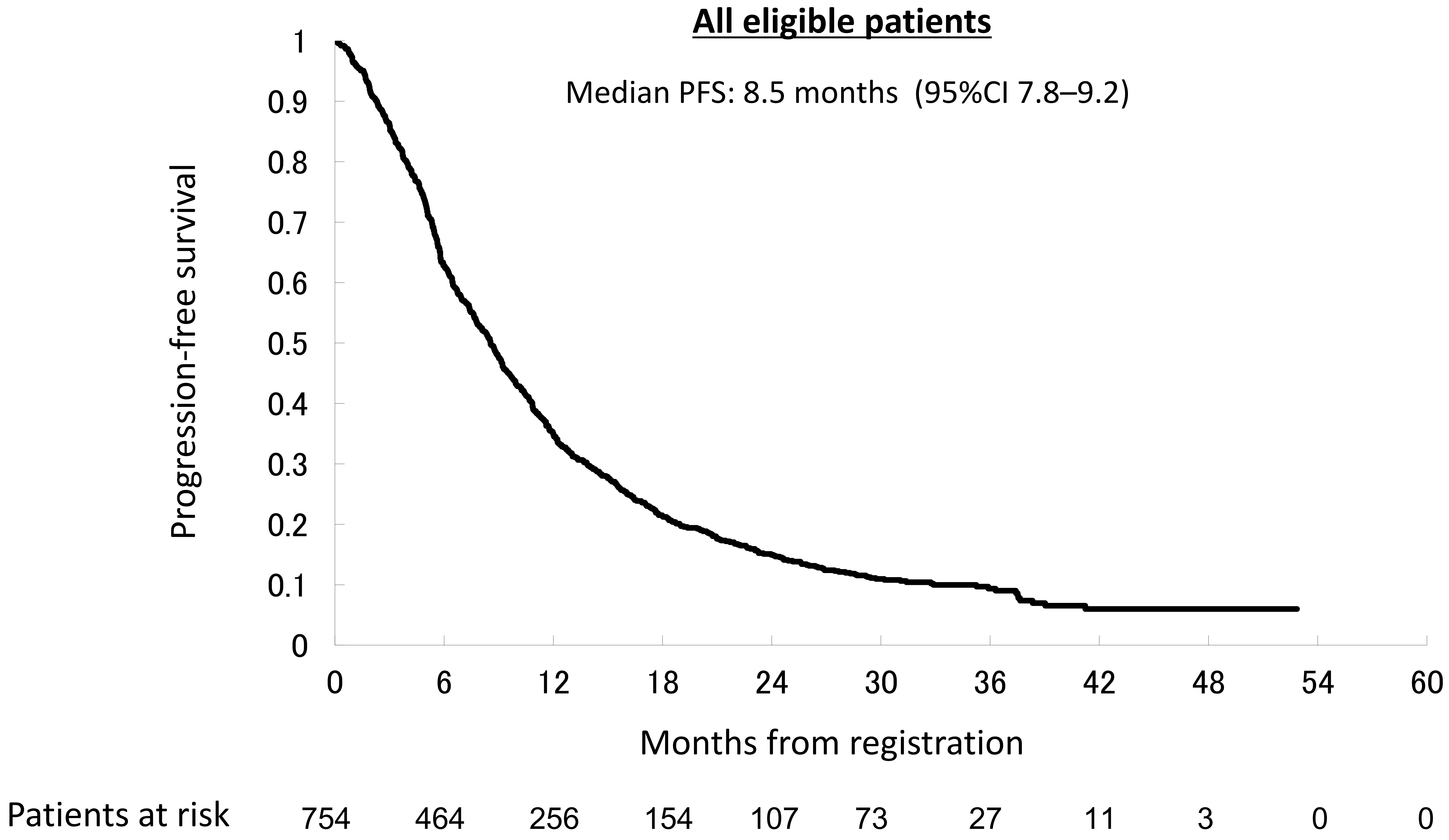

Supplementary Figure 2a

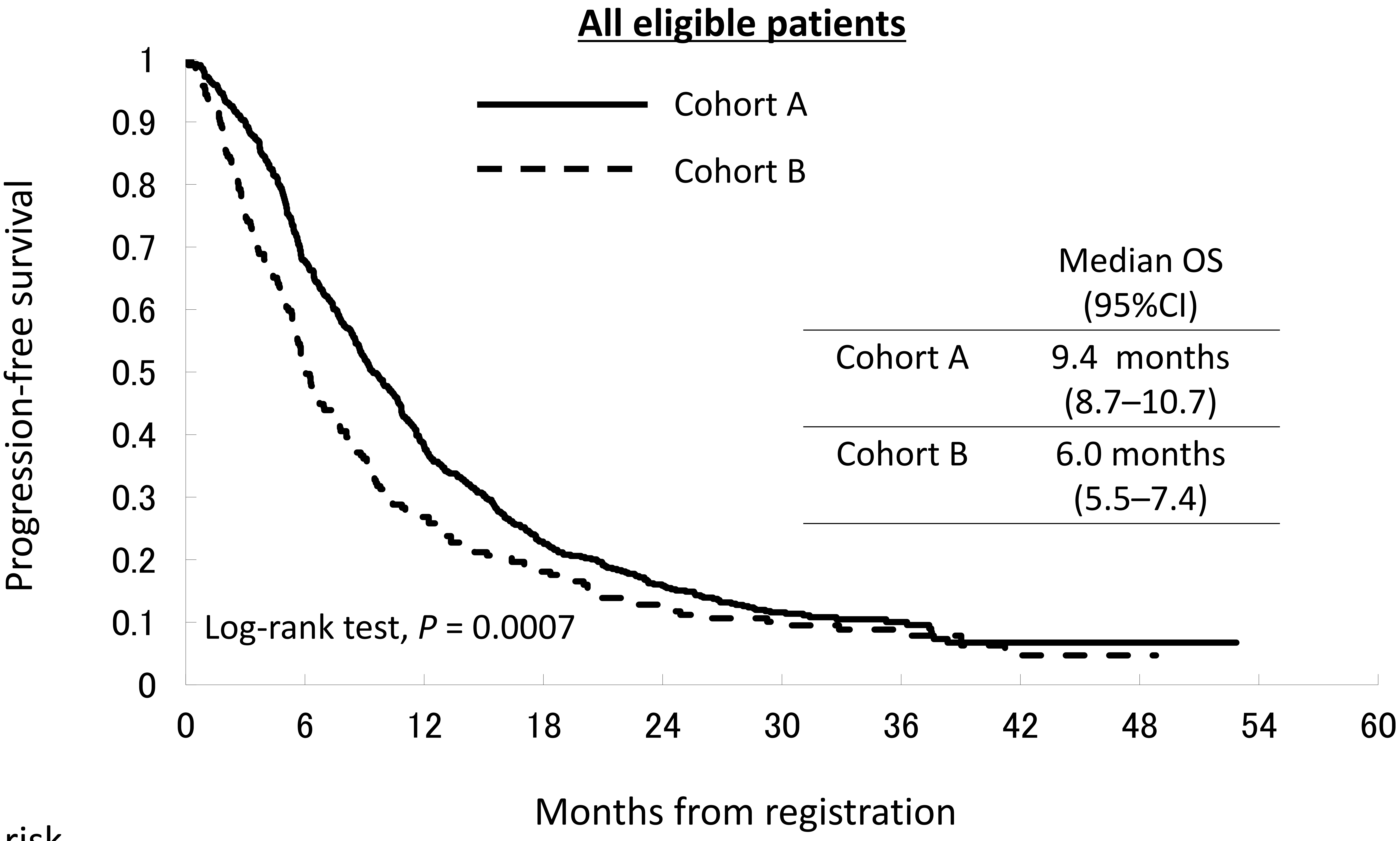

Patients at risk

Cohort A	539	360	203	119	83	56	21	8	1	0	0
Cohort B	215	104	53	35	24	17	6	3	2	0	0

Supplementary Figure 2b

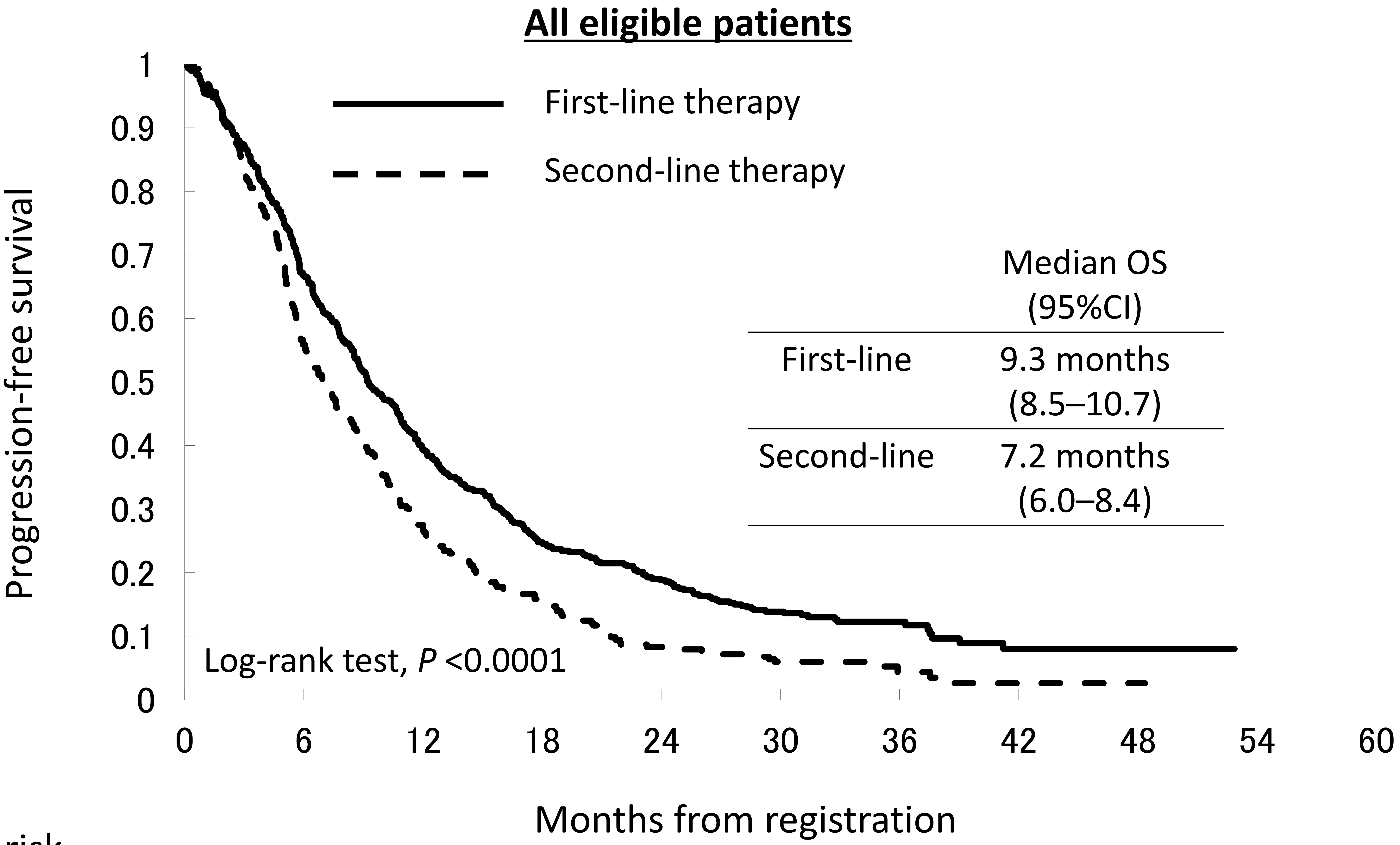

Patients at risk

First-line therapy	478	312	182	113	85	58	22	9	2	0	0
Second-line therapy	276	152	74	41	22	15	5	2	1	0	0

Supplementary Figure 2c

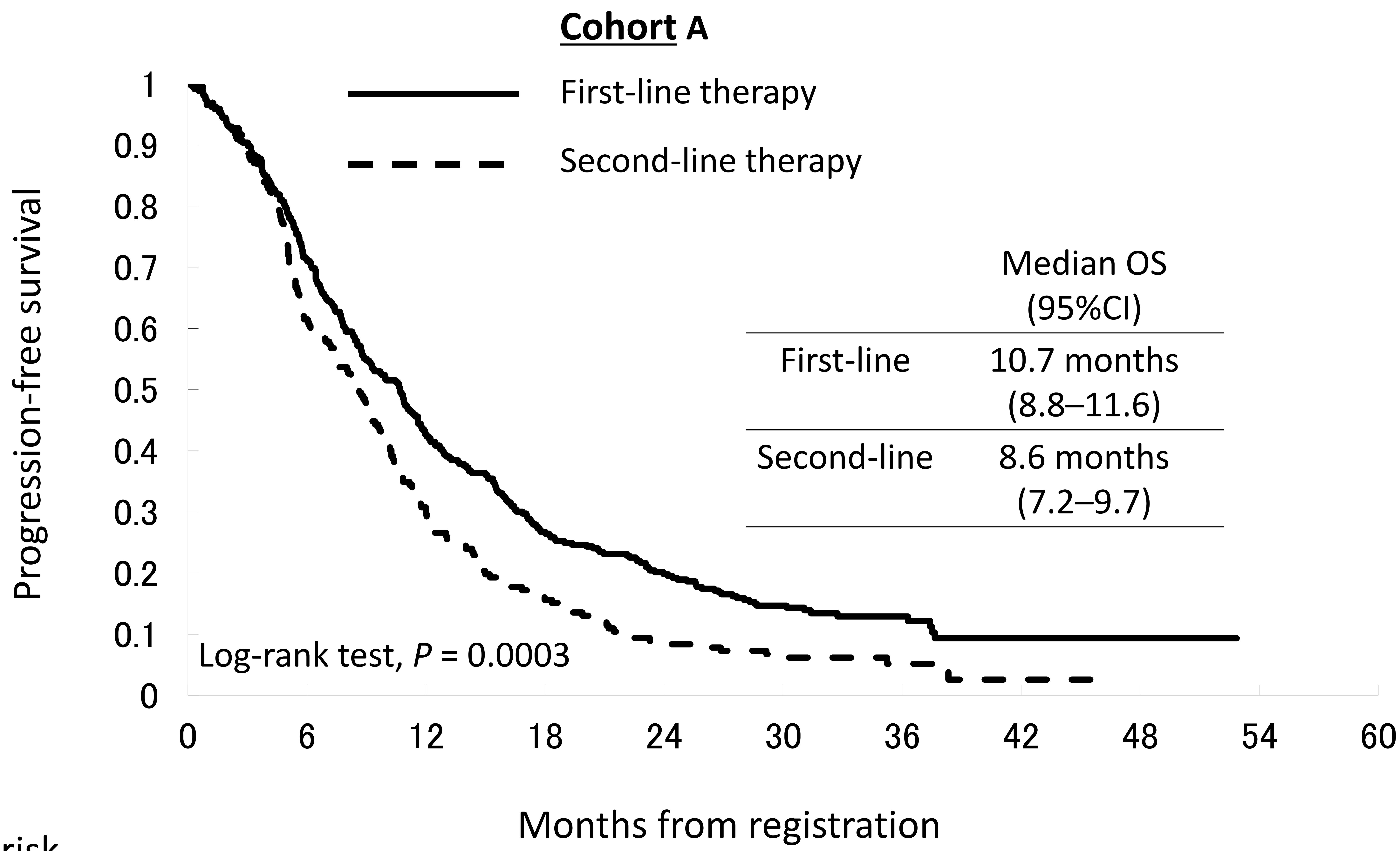

Patients at risk

First-line therapy	345	242	144	89	66	45	17	7	1	0	0
Second-line therapy	194	118	59	30	17	11	4	1	0	0	0

Supplementary Figure 2d

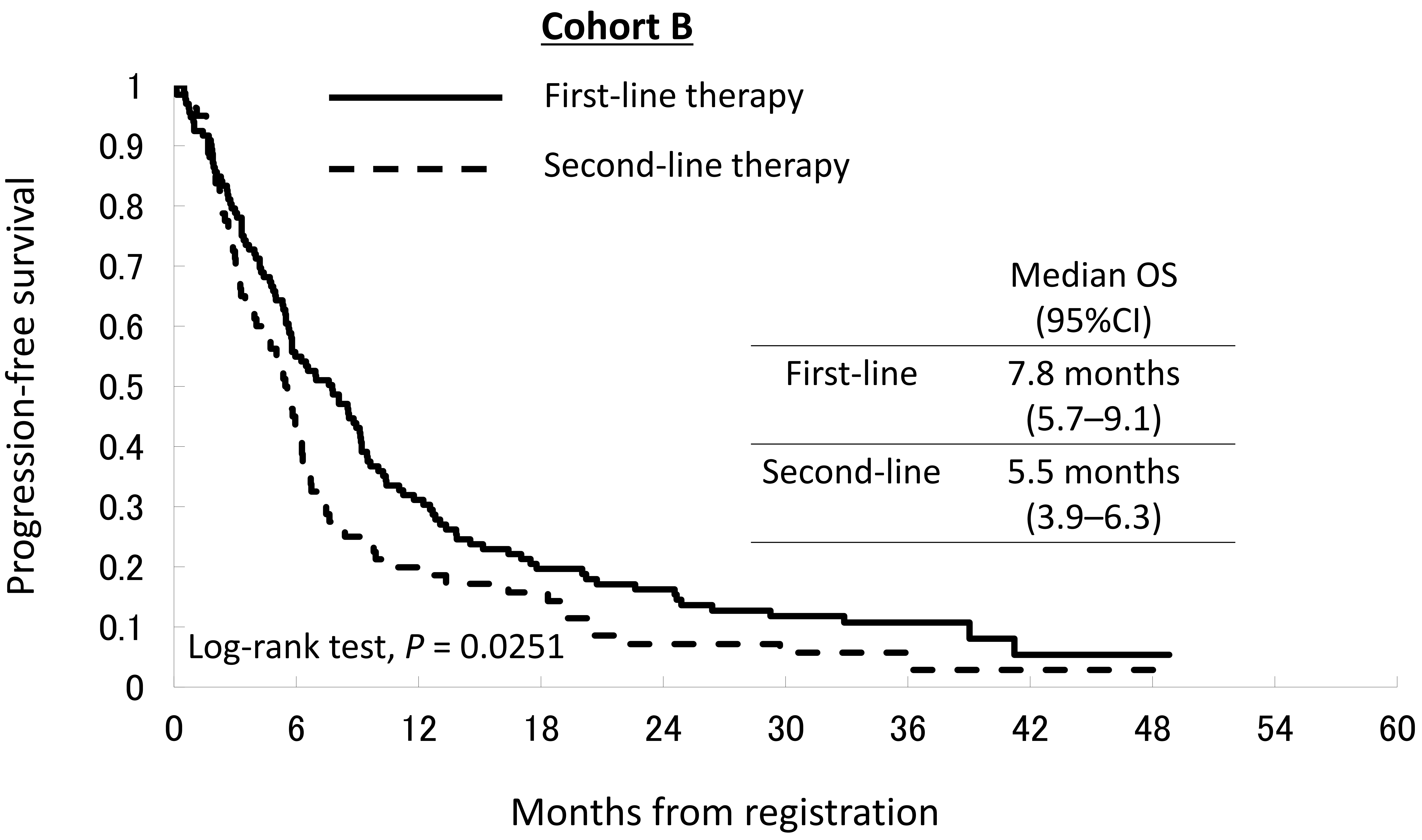

Patients at risk

First-line therapy	133	70	38	24	19	13	5	2	1	0	0
Second-line therapy	82	34	15	11	5	4	1	1	1	0	0

Supplementary Figure 2e

Figure legends

Supplementary Fig. 1 Patient disposition. Cohorts A and B comprised patients with hormone receptor–positive breast cancer and triple-negative breast cancer, respectively.

Supplementary Fig. 2 Progression-free survival in the eligible patient population: **a**, all eligible patients; **b**, cohort A (patients with hormone receptor–positive breast cancer) versus cohort B (patients with triple-negative breast cancer); **c**, all eligible patients receiving first-line versus second-line therapy; **d**, first-line versus second-line therapy in cohort A; **e**, first-line versus second-line therapy in cohort B.
